# Brachytherapy Drainage Catheter and Chemotherapy for Unresectable Pancreatic Carcinoma Combined with Obstructive Jaundice

**DOI:** 10.3389/fonc.2022.941336

**Published:** 2022-07-14

**Authors:** Dechao Jiao, Kaihao Xu, Gauri Mukhiya, Yiming Liu, Kunpeng Wu, Zongming Li, Jianzhuang Ren, Xinwei Han

**Affiliations:** Department of Interventional Radiology, First Affiliated Hospital of Zhengzhou University, Zhengzhou, China

**Keywords:** malignant biliary obstruction, ^125^I brachytherapy, pancreatic carcinoma, stent, chemotherapy

## Abstract

**Background:**

Most patients with advanced pancreatic cancer do not have the chance to undergo surgery or chemotherapy because of their poor conditions. Biliary drainage is a palliative treatment to restore liver function and alleviate jaundice, but most patients still face the risk of biliary obstruction in the short term after operation. The purpose of this study is to evaluate the efficacy and safety of brachytherapy drainage catheter (BDC)-combined chemotherapy in the treatment of pancreatic cancer complicated with obstructive jaundice.

**Patients and Methods:**

From November 2017 and May 2019, 48 patients underwent the BDC or conventional drainage catheter (CDC) intervention with chemotherapy. The outcomes/endpoints analyzed were technical and clinical success, early complications, stent patency period, and survival.

**Results:**

The technical and clinical success rates in both groups were 100%, and the early complication rates were not significantly different (P = 0.43). The median stent patency in the BDC group was significantly longer than that in the CDC group (7.8 ± 1.5 vs. 5.7 ± 0.7 months, P = 0.001), and the median overall survival period in the BDC group was prone to significant difference than that in the CDC group (9.4 ± 4.0 vs. 8.2 ± 0.3 months, P = 0.089).

**Conclusion:**

The findings of this study show that BDC with chemotherapy was associated with better stent patency and survival. However, since the sample size was very small, large randomized controlled multicenter studies are needed to further evaluate the long-term survival effects of BDC in patients with advanced pancreatic carcinoma combined with obstructive jaundice.

## Introduction

Pancreatic cancer (PC) is characterized by occult onset, high malignancy rate, and poor prognosis (1). Pancreaticoduodenectomy combined with local lymph node dissection is still a radical treatment for pancreatic head cancer. Most patients who are in the advanced stage of PC have biliary tract obstruction and jaundice symptoms, and therefore, one of the aims of treatment is to restore the structure and function of the biliary tract, alleviate jaundice, and improve the patients’ nutritional status and quality of life (2). Although surgical resection is the best strategy, more than 70% of patients diagnosed with PC combined with biliary obstruction are not eligible for radical surgery on account of local invasion, metastasis, poor condition, old age, etc. (3) The alternatives for these patients are limited and include palliative biliary drainage and subsequent chemotherapy. Biliary drainage is the preferred palliative approach to restore liver function and decrease complications in these patients, but most patients who undergo this procedure develop jaundice again 4–6 months later due to tumor ingrowth and overgrowth (4). Although many types of biliary stents, such as partially or fully covered stents, or coated stents with better patency and better efficiency have been developed, the clinical results are still unsatisfactory (5). Therefore, this method might need more improvements in the future.

In PC patients who have undergone treatment for biliary obstruction, once the damaging effect of cholestasis on hepatocytes is gradually reduced and liver function is improved, systemic chemotherapy or radiotherapy is administered (6). Although three-dimensional conformal radiotherapy is promising for cancer treatment, it has limited effects in PC on account of the unique structure and position of the pancreas (7). Additionally, in advanced PC cases with obstructive jaundice, the radiation dose that can be used is limited on account of the poor condition of the patient and limited liver function (8, 9). A suitable alternative is the use of ^125^I radioactive seeds that can continuously release low-dose γ-rays to inhibit cancer cells and promote apoptosis. Many animal and clinical studies have confirmed that ^125^I brachytherapy can obviously increase the local control rate and improve long-term survival (10–13). At present, the most commonly tools for the permanent implantation of radioactive ^125^I seeds is CT or US guidance, which requires percutaneous trans-stomach puncture to the tumor located at the head of pancreas frequently, which is a high-risk treatment due to the complex surrounding vessels. If the patient has advanced PC with obstructive jaundice, it is necessary to solve the jaundice before chemotherapy. Thus, whether the percutaneous drainage can be combined with ^125^I brachytherapy is a valuable clinical scientific topic; what is more, transbiliary intralunimal ^125^I brachytherapy can decrease the puncture-associated complications. In 2017, our research team also reported that brachytherapy with an ^125^I strand can be used for treating malignant biliary obstruction and prolonging stent patency (14). However, if a biliary stent is placed before an ^125^I strand is placed, the tumor could be pushed outside by the stent and the radiation might not cover the entire tumor volume. Therefore, at our center, we explored the use of two ^125^I strands along with the biliary stent in order to meet the goals of reducing the tumor and decreasing stent ingrowth and extending its patency. We designed an internal–external biliary drainage catheter (BDC) with three cavities to fulfil the goals of drainage and therapy. Bile is drained out *via* the central cavity, while both side cavities contain radioactive ^125^I seeds for brachytherapy. Once the jaundice is treated, gemcitabine combined with cisplatin is the standard chemotherapy for PC. The purpose of this study is to evaluate the safety and effectiveness of BDC followed by subsequent chemotherapy for the treatment of advanced PC and biliary obstruction.

## Materials and methods

### Study Design And Patients

**Figure 1:**
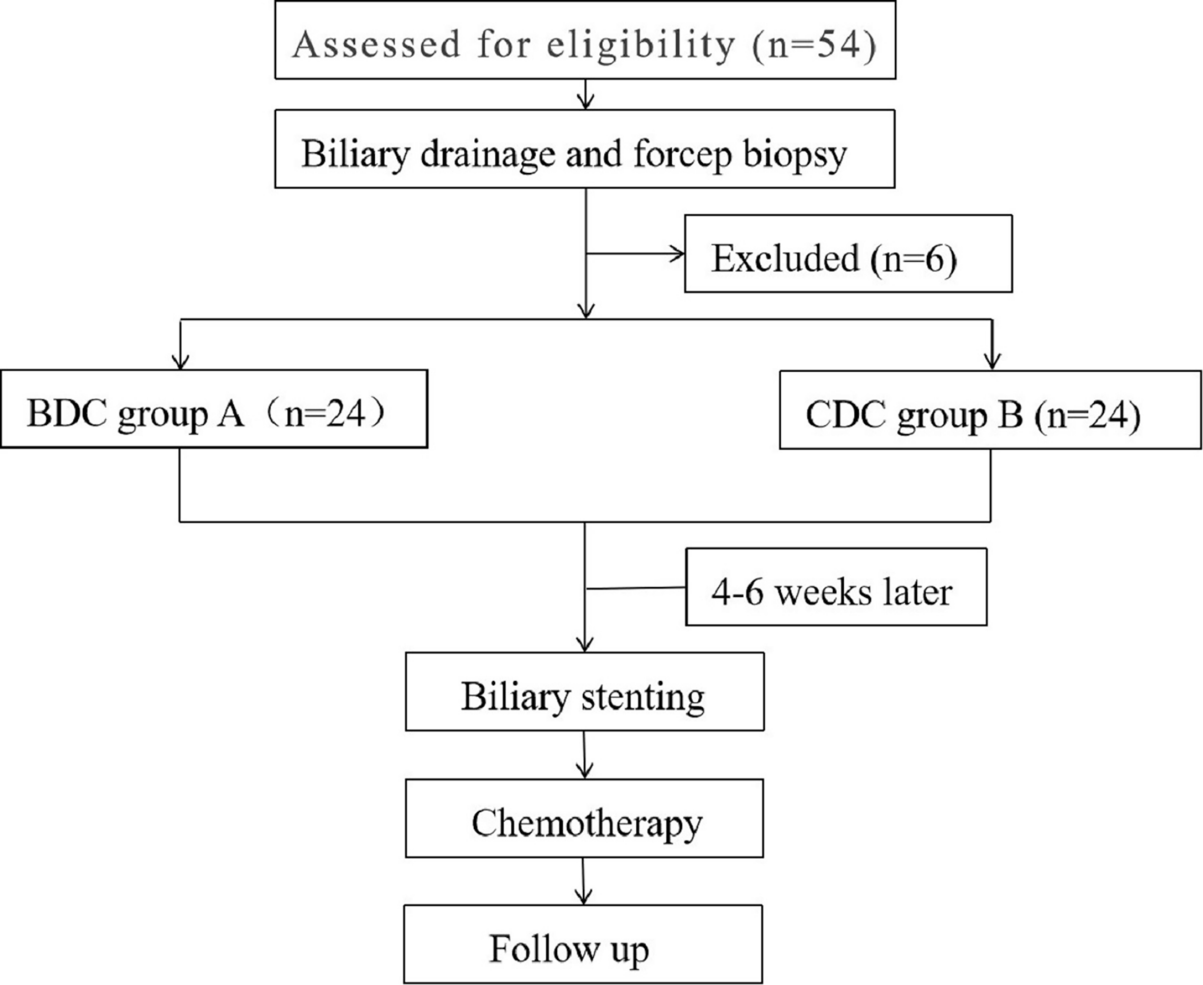
Workflow of the study.

All procedures followed were in accordance with the ethical standards of the responsible committee on human experimentation (institutional and national) and with the Helsinki Declaration of 1964 and later versions. With the approval of the ethics committee of our hospital, 48 patients with advanced-stage PC accompanied by biliary obstruction underwent the BDC or conventional drainage catheter (CDC) intervention with chemotherapy. The inclusion criteria for the study were as follows: 1) advanced-stage PC with confirmed pathology accompanied by biliary obstruction, 2) age between 18 and 75 years, 3) Eastern Cooperative Oncology Group (ECOG) score of 0–2, 4) unresectability or refusal to undergo conventional surgery, and 5) normal or grade I according to The New York Heart Association (NYHA) functional classification; forced expiratory volume in 1 s ≧70%; creatinine levels 20–115 µml/l. The exclusion criteria were 1) uncontrollable ascites, 2) severe coagulation dysfunction, 3) ECOG score of 3–4, 4) non-malignant stenosis, 5) extensive hepatic metastases, and 6) the lack of appropriate approaches for percutaneous puncture. The workflow is listed in **Figure 1**.

### Procedures

**Figure 2:**
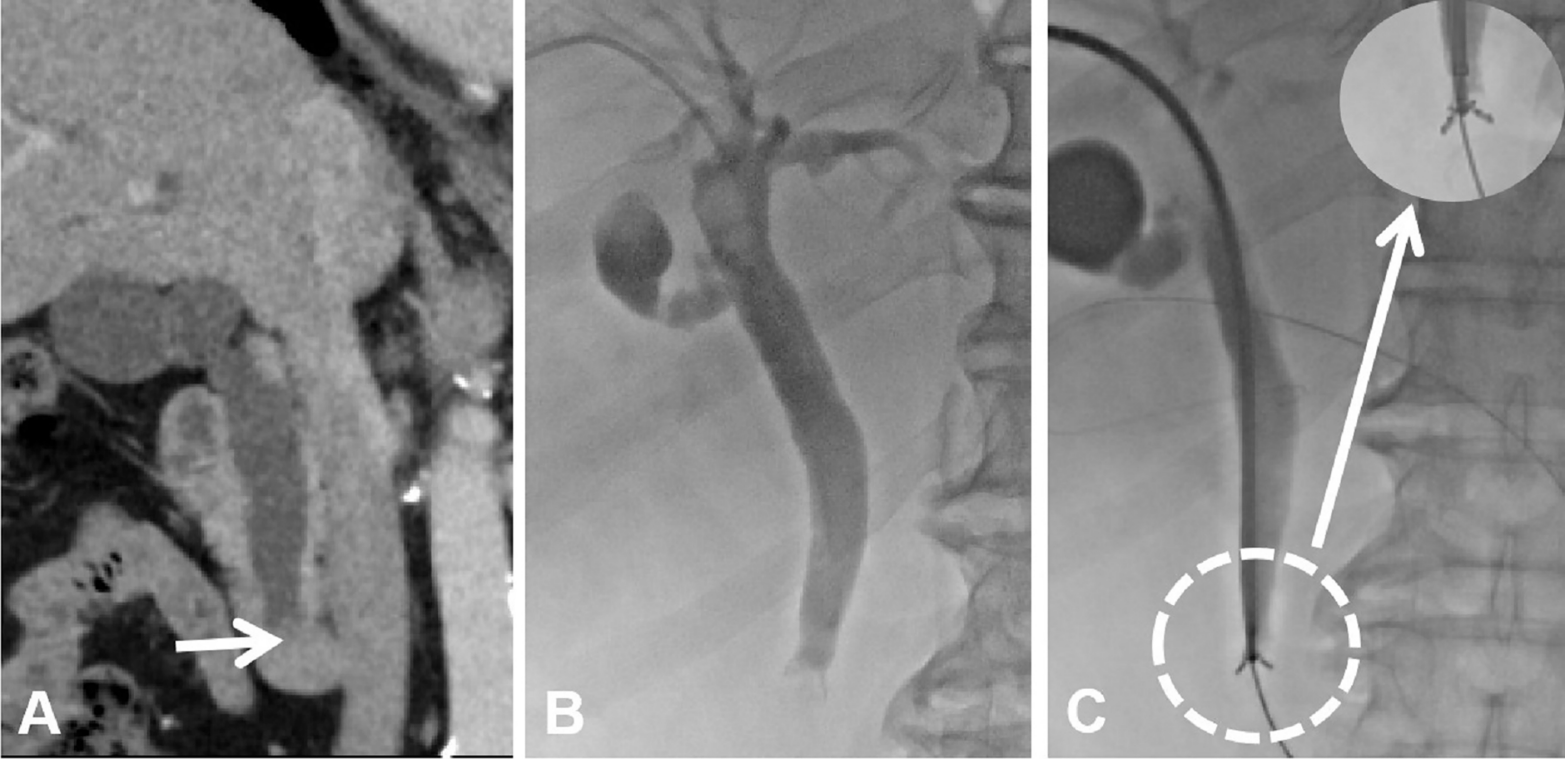
Imaging and biopsy of the biliary obstruction. **(A)** Coronal enhanced CT scan showing the lower biliary obstruction caused by PC. **(B)** Percutaneous transhepatic cholangiography was performed to visualize the location and degree of biliary obstruction. **(C)** Forceps biopsy was performed at the site of the biliary obstruction *via* a 9-F long sheath.

**Figure 3:**
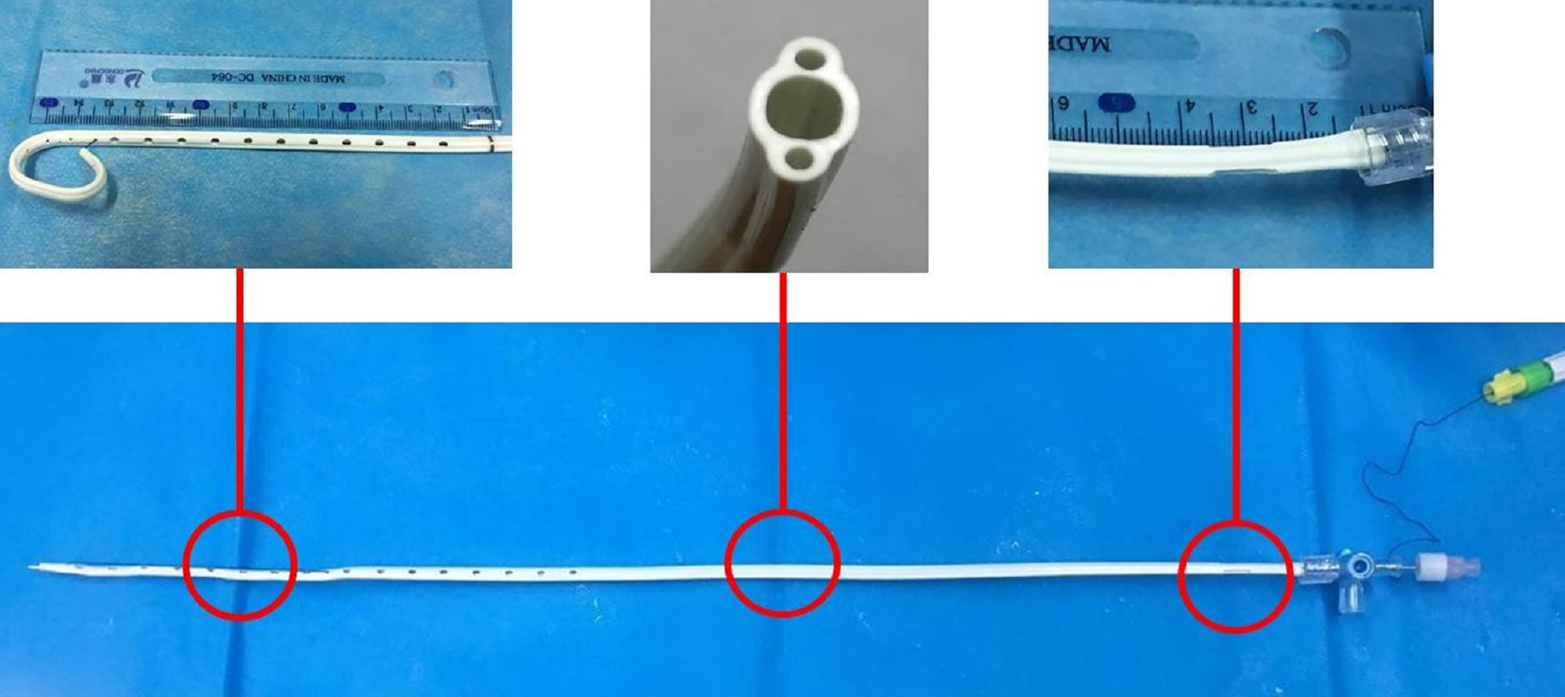
Brachytherapy drainage catheter with three separate cavities: the central cavity has drainage function, and the lateral cavities carry radioactive ^125^I seeds.

**Figure 4:**
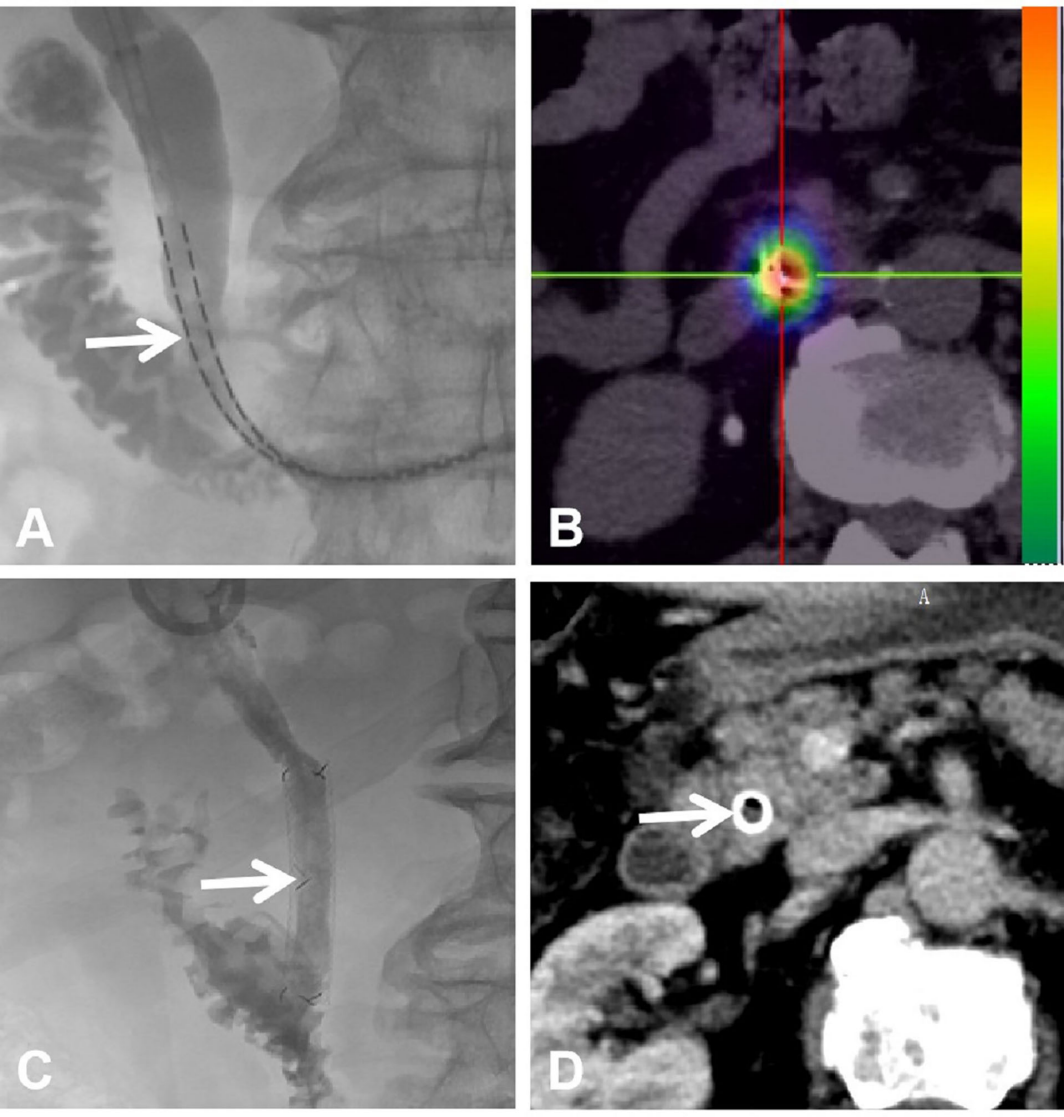
Placement of the brachytherapy drainage catheter and stent patency after 9 months. **(A)** The brachytherapy drainage catheter was inserted across the PC to the duodenum. **(B)** SPECT/CT scan showing radiation emitted by ^125^I brachytherapy within the PC. **(C)** Biliary stent after brachytherapy showing stent patency. **(D)** CT scan showing stent patency at the 9-month follow-up.

Enhanced MR (including magnetic resonance cholangiopancreatography, MRCP) or CT were performed before interventional procedures. The patient was required to lay supine on the table for digital subtraction angiography (Artis Zeego; Siemens, Erlangen, Germany). Basic anesthesia was maintained by intravenous infusion of digoxin (5 mg) and dexmedetomidine hydrochloride (200 µg), and 2% lidocaine (5 ml) was used at the puncture site. Under color Doppler ultrasound guidance, a 21-G puncture needle (PTC; Cook Inc., Bloomington, IN, USA) was used to puncture the bile duct. Then, a 0.018-inch platinum guidewire was introduced and replaced with a 6F sheath to perform cholangiography for measuring the extent of stenosis. With the help of a guidewire, a 5-F catheter was passed through the occlusion/stenosis part to the duodenum, and it was replaced with a super-stiff guidewire (Boston Scientific, Natick, MA, USA). Next, a 9F catheter (length, 23 cm; Cordis, Santa Clara, CA, USA) was advanced along the guidewire until it reached the occlusion/stenosis part, and forceps biopsy was performed as reported in our previous paper (**Figure 2**) (15). In order to decrease the BDC implantation resistance, a 10-F short sheath (Cook, USA) was used to expand the puncture access. A 10F BDC (Tuoren, Henan, China) (**Figure 3**) with three separate cavities was used in the BDC group. The central cavity had 12 holes for bile drainage, and the side cavities (internal diameter, 0.85 mm) were loaded with ^125^I radioactive seeds (size: 0.8 × 4.5 mm) emitting low-energy 35.5 keV γ rays with a half-life of 59.6 days (Biopharmaceutical Co. Ltd., Tianjin, China). The amount of radioactivity contained within each seed was 25.9 MBq (0.7 mCi). Based on the length of the biliary stenosis segment measured *via* angiography and previous irradiation dose experience (14), the following formula was used to calculate the number of seeds: 2 × (the stenosis length [mm] + 40 mm/4.5). The position of the seeds was verified by SPECT/CT (**Figure 4**) within 4 days after BDC placement, and the estimated radiation dose at 5 mm from the catheter was calculated with the help of a computerized treatment plan system (TPS). In the control/CDC group, a 10.2-F external–internal biliary catheter (Cook, USA) was placed across the stenosis for drainage (**Figure 4**).

After 4–6 weeks of treatment in both groups, the bilirubin decreased to normal, liver function recovered, and the general condition of the patients was significantly better than that before treatment. In both groups, the catheters were replaced with biliary stents (**Figure 4**). Biliary self-expandable nitinol stents from Tae-woong (Seoul, Korea) and Micro-tech (Nanjing, China) were used (diameter = 10 mm, length = 50–80 mm). The biliary stent was deployed across the stenosis/occlusion zone, and poor stent expansion was amended with balloon catheter expansion (diameter = 6–8 mm; Boston Scientific, Natick, MA, USA). Then, a standard chemotherapy regimen with gemcitabine and cisplatin was administered to all patients.

### Endpoints and Follow-Up

The study endpoints were stent patency, technical feasibility and safety of the procedure, early (within 1 month) complications, and overall survival. Technical success was defined as successful placement of CDC or BDC and the metal stent. Clinical success was defined as a decrease in the bilirubin level by at least 75% of the baseline value, and disappearance or significant alleviation of jaundice-related fever, poor appetite, and weakness. All complications were defined according to the Society of Interventional Radiology (SIR) Clinical Practice Guidelines which are divided into early (≤30 days) and late (>30 days) complications. The stent patency period was considered as the duration from the initial stent placement to the day of recurrence of malignant obstruction symptoms with an obvious increase in bilirubin. The follow-up time was measured as the time from placement of the drainage tube until the end of the study or the patient’s death. Blood examinations and medical imaging were performed at 1 and 3 months after treatment and every 3 months thereafter.

### Statistical Analysis

The numerical data are expressed as mean ± standard deviation. An independent t-test or chi-square test was used to analyze the primary characteristics (SPSS 17.0 software; SPSS Inc., Chicago, USA). Wilcoxon signed-rank test was used to examine the difference in the data before and after the procedure within the same group, while the Mann–Whitney U-test was used to examine differences in variance between the BDC and CDC group. The Kaplan–Meier method was used to examine stent patency and survival. Complications between groups were compared with the Fisher exact test. P values < 0.05 were considered to indicate statistical significance.

## Results

Primary characteristics such as age, sex, biliary stenosis length, ECOG score, and blood test results were similar between the two groups (P > 0.05) (**Table 1**). The prescription dose was 100 Gy, and the mean radiation dose in the BDC group was 83.5 ± 5.9 Gy (range, 74.1–96.4 Gy).

**Table 1:** Patient characteristics.

Characteristics	BDC+Chemotherapy	CDC+Chemotherapy	P value
Mean age (years)	58.7 ± 15.6	61.6 ± 12.0	0.46^*^
Sex			0.24^#^
Female	16	12	
Male	8	12	
Time to diagnosis	22.5 ± 9.0	26.3 ± 11.5	0.21^*^
Biliary stenosis length (mm)	31.2 ± 5.6	29.9 ± 5.5	0.44^*^
Local tumor diameter (mm)	27.0 ± 10.1	25.8 ± 8.6	0.65^*^
Clinical stage			0.81^#^
I	2	3	
II	8	7	
III	11	9	
IV	3	5	
ECOG score			0.19^#^
1	11	15	
2	13	9	
CA19-9 (U/mL)	723.5 ± 697.8	683.2 ± 693.2	0.84^*^
Laboratory values			
White blood cell (×109/L)	6.2 ± 1.4	6.4 ± 1.7	0.62^*^
Hemoglobin (g/L)	118.5 ± 29.1	127.4 ± 18.0	0.21^*^
Serum bilirubin (µmol/L)	180.5 ± 53.7	169.4 ± 58.5	0.50^*^
Direct bilirubin (µmol/L)	136.7 ± 46.8	134.9 ± 50.5	0.90^*^
Albumin (g/L)	38.7 ± 2.7	39.0 ± 2.9	0.71^*^
Alanine aminotransferase (U/L)	105.9 ± 44.5	91.9 ± 40.6	0.26^*^
Alkaline phosphatase (U/L)	642.8 ± 335.4	614.8 ± 297.0	0.76^*^
Chemotherapy course			0.73^#^
2–4	18	19	
4-6	6	5	

Data shown are the number or mean ± SD; ^*^ Independent samples t-test was used; ^#^ χ^2^ test was used.

With regard to the outcome, 100% technical and clinical success was achieved in all 48 cases. In both groups, the bilirubin levels of all the patients decreased significantly within 4–5 weeks, and there were significant improvements in the patients’ appetite, fever, and other conditions, as well as the ECOG score. The bilirubin level at posttreatment 1 month in the BDC (68.2 ± 20.0 µmol/l; range, 36.9–102.3 µmol/l) and CDC group (64.4 ± 16.8 µmol/l; range, 38.1–110.3 µmol/l) was significantly lower than the corresponding baseline values (BDC: 180.5 ± 53.7 µmol/l; range, 94.1–340.1 µmol/l) (CDC: 169.4 ± 16.8 µmol/l; range, 93.6–342.1 µmol/l). However, there was no significant difference between the two groups with regard to the total bilirubin, direct bilirubin, alanine aminotransferase, and hemoglobin values (P > 0.05) (detail information is list in **Table 2**).

**Table 2:** Laboratory values and early complications in both groups.

Characteristics	BDC+Chemotherapy	CDC+Chemotherapy	P value
Serum bilirubin level			0.29^#^
Before	180.5 ± 53.7	169.4 ± 16.8	
After	68.2 ± 20.0	64.4 ± 16.8	
P value	0.00^*^	0.00^*^	
Direct bilirubin			0.64^#^
Before	136.7 ± 46.8	135.0 ± 38.4	
After	42.4 ± 15.0	38.4 ± 11.5	
P value	0.00^*^	0.00^*^	
Alanine aminotransferase			0.22^#^
Before	105.9 ± 44.5	91.9 ± 40.6	
After	49.8 ± 14.0	47.4 ± 13.3	
P value	0.00^*^	0.00^*^	
Hemoglobin			0.36^#^
Before	123.1 ± 18.3	127.4 ± 18.0	
After	125.1 ± 18.4	126.6 ± 15.4	
P value	0.73^*^	0.94^*^	
Early complications			0.43^ψ^
Cholangitis	2	4	
Pancreatitis	1	0	
None	21	20	

*Difference in the values before and after the intervention in the same group (Wilcoxon signed-rank test); ^#^Difference in the variance between the two groups (Mann–Whitney U-test); ψ Fisher’s exact test.

**Figure 5:**
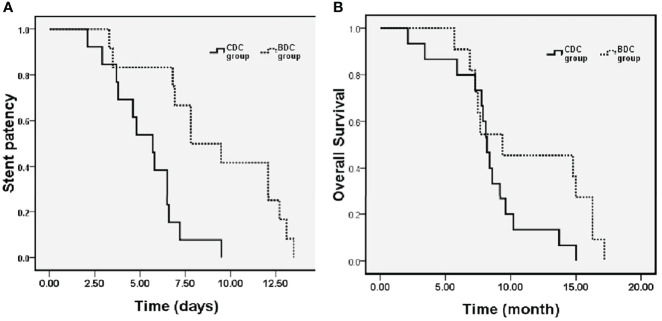
Kaplan–Meier curves showing cumulative stent patency **(A)** and overall survival **(B)** of BDC and control groups.

During the mean follow-up period of 10.2 months (range, 3.4–17.5 months), stent occlusion occurred in 13 cases (54.2%) in the CDC group and 12 cases (50%) in the BDC group. Further, reintervention was required in eight cases in the CDC group (four stents and four drainage catheters) and seven cases (three stents and four drainage catheters) in the BDC group. The median and mean stent patency periods were 7.8 months (95% CI = 4.9–10.7) and 9.0 months (95% CI = 7.0–11.1), respectively, in the BDC group, and they were 5.7 months (95% CI = 4.3–7.1) and 5.4 months (95% CI = 4.3–6.4), respectively, in the CDC group. Stent patency were significantly better in the BDC group than in the CDC group (P > 0.05) (**Figure 5**). The median and mean survival was 9.4 months (95% CI = 1.5–17.3) and 11.3 months (95% CI = 8.6–14.0), respectively, in the BDC group, and 8.2 months (95% CI = 7.6–8.8) and 8.4 months (95% CI = 6.7–10.0), respectively, in the CDC group. The BDC group is prone to significant difference compared with the CDC group (9.4 ± 4.0 vs. 8.2 ± 0.3 months, P = 0.089). There were 15 (62.5%) deaths in the CDC group that occurred on account of disease progression (n = 8), hepatic failure (n = 5), and recurrent gastrointestinal bleeding (n = 2). Further, there were 11 (45.8%) deaths in the BDC group that occurred on account of disease progression (n = 7), hepatic failure (n = 3), and severe lung infection (n = 1).

With regard to complications, there were no cases of severe early complications such as bleeding, abscess formation, and biliary rupture. There were only three cases (12.5%) and four cases (16.7%) of early complications in the BCD and CDC groups, respectively (P = 0.43) (**Table 2**).

## Discussion

In this study, we have explored the effectiveness of a newly designed BDC that meets the purpose of biliary drainage and intraluminal brachytherapy. We have compared this device with the conventionally used drainage catheter that does not have a brachytherapy function. In our patient groups, both catheters were associated with a technical and clinical success rate of 100% and a complication rate of 12.5%. Further, with BDC, the mean bilirubin level decreased significantly from 180.5 μmol/l (before treatment) to 68.2 μmol/l (after treatment); this was similar to the drainage effect of CDC. This implies that the three-lumen structure of BDC has excellent drainage ability and a reasonable complication rate, despite its non-cylindrical shape.

In the present study, the median and mean stent patency periods were 7.8 and 9.0 months in the BDC group (5.7 and 5.4 months for CDC group, respectively). These values are encouraging as they were significantly greater than those in the CDC group. The prolonged stent patency of BDC might be attributable to the ^125^I seeds, as they prohibit tumor growth and thereby decrease tumor in-growth through the stent mesh after biliary stenting, thus extending stent patency (16). In 2009, Liu Y et al. (17) firstly improved the common biliary plastic stent structure by designing the lateral channel (0.7 mm) in the plastic stent wall for loading ^125^I seed and discussed its application in malignant biliary obstruction *via* retrograde cholangiopancreatography approach. Unfortunately, the smaller central drainage cavity (1.8 mm) is easy to cause blockage and inhibit its further clinical use. Chen et al. reported that a single ^125^I strand can be used to treat malignant biliary obstructions; further, they also reported that the stent patency of the brachytherapy group (mean, 10.2 months) was longer than that of the conventional stent group (mean, 7.2 months) (18). However, the problem was that the ^125^I strand was pressed against the side wall outside the stent leading to irradiation dose eccentricity. Moreover, the ^125^I strand cannot be removed if there are severe early complications. Zhu et al. developed a new type of device that included a brachytherapy stent with ^125^I seeds and a conventional stent and found that it had long-term patency (19). However, deployment of their stent is complex, especially in cases of severe occlusion. Despite this, the clinical result was satisfactory, as the stent patency and overall survival were better than that of the conventional stent. Similar to the present study, these previous studies also demonstrate that ^125^I brachytherapy is feasible and effective for malignant biliary obstruction. However, there are some differences, and this is probably attributable to the patient selection process: the present study included patients with pancreatic cancer who had low bile duct obstruction (mean tumor diameter, 27 mm), and the other previous study used a more complicated classification that included bile duct cancer, liver metastasis, liver cancer, liver hilar metastasis, etc.

In the present study, there was no significant difference in the complication rate between the two groups. This means that BDC had a high clinical application value. The mean estimated radiation dose was 83.5 Gy, which was similar to the mean dose reported in Chen et al.’s study (87.5 Gy) 18. The therapeutic dose with BDC is satisfactory, given that Chen’s study used single-strand brachytherapy and permanent implantation and BDC employs temporary implantation of a double-stranded brachytherapy stent (4–6 weeks). External irradiation for PC must pay more attention to the gastrointestinal tract, liver, and portal vein, because of high-dose injury limitation, and gastrointestinal tract ulcer occurrence is easy if the irradiation dose exceeds 50 Gy (20). On the other hand, there is no worry about ^125^I brachytherapy damage to the surrounding organs, because 80% dose is delivered within 1 cm with a consistent, low-dose-rate-delivering way (21). Considering that the tumor size has a great impact on the local dose accumulation of ^125^I brachytherapy, for tumors larger than 3 cm, it is theoretically recommended that the BDC combined with percutaneous ^125^I seed implantation may better control the local tumor. Further, in this study, the long-term survival was prone to be significantly longer in the BDC group. However, given the small sample size, further follow-up is needed to confirm this finding.

The main drawbacks of the BDC was its ellipse shape which will increase the BDC implantation resistance, the integrated cylindrical three-cavity drainage design may be the best choice. This study also has several limitations. The main limitation was that the sample size was too small to provide sufficient statistical power, especially with regard to the overall survival data. Additionally, it was difficult to evaluate tumor response because the tumor was pressed by the stent. Finally, the ^125^I seed dose measured was not accurate because there is no space between the seeds in BDC and they are not uniformly distributed. The measurement and distribution of the seeds require further research.

## Conclusion

In conclusion, this study demonstrates the feasibility and safety of BDC for the treatment of PC with biliary obstruction. In the future, multiple-center studies are needed to clarify its effectiveness and long-term effects.

## Data Availability Statement

The raw data supporting the conclusions of this article will be made available by the authors, without undue reservation.

## Ethics Statement

The studies involving human participants were reviewed and approved by 2017-KY-355. The patients/participants provided their written informed consent to participate in this study.

## Author Contributions

DJ and KX: primary investigator, involved in study planning, data collection, data analysis and interpretation, and manuscript writing. GM, YL, KW, and ZL: involved in study planning, data collection, data analysis and interpretation, and proofreading of manuscript. JR and XH: involved in study planning, data collection, data analysis and interpretation, manuscript writing, and proofreading of manuscript. All authors agree to be accountable for the content of the work.

## Conflict of Interest

The authors declare that the research was conducted in the absence of any commercial or financial relationships that could be construed as a potential conflict of interest.

## Publisher’s Note

All claims expressed in this article are solely those of the authors and do not necessarily represent those of their affiliated organizations, or those of the publisher, the editors and the reviewers. Any product that may be evaluated in this article, or claim that may be made by its manufacturer, is not guaranteed or endorsed by the publisher.
